# Presentation of endometrial vascular dystrophy under narrow-band imaging: Two case reports and literature review

**DOI:** 10.1097/MD.0000000000041807

**Published:** 2025-07-04

**Authors:** QiYin Zhou, HaiHong Cui, WenDi Han, JinCheng Huang

**Affiliations:** a Yanhe Tujia Autonomous County People’s Hospital, Tongren, Guizhou Province, China; b Dongguan SongShan Lake Central Hospital, Dongguan, Guangdong Province, China.

**Keywords:** capillary loop, case report, endometrial polyps, endometrial vascular dystrophy, hysteroscopy, narrow-band imaging, NBI

## Abstract

**Rationale:**

Endometrial vascular dystrophy is a rare and poorly understood pathological condition characterized by tortuous and dilated blood vessels visible under hysteroscopy. Diagnosing this condition is challenging due to its potential resemblance to more severe lesions, such as endometrial hyperplasia or carcinoma. Narrow-band imaging (NBI) technology enhances the visualization of superficial vascular structures, offering a new perspective for diagnosis. This study aims to explore the application value of NBI technology in identifying endometrial vascular abnormalities, thereby improving diagnostic accuracy and differentiating this condition from more serious pathologies.

**Patient concerns:**

Case 1 is a 32-year-old woman presenting with vaginal bleeding for 8 days and a history of intermittent bleeding for 2 months. Case 2 is a 47-year-old woman with increased menstrual flow and prolonged periods for the past 2 months. Hysteroscopy revealed endometrial vascular dystrophy in both cases, with the presence of endometrial polyps.

**Diagnoses:**

Both patients were diagnosed with endometrial vascular dystrophy and endometrial polyps.

**Interventions:**

Hysteroscopy in both cases revealed a multitude of curved and dilated cord-like capillary loops, along with endometrial polyps. Targeted biopsies and polyp excision were performed.

**Outcomes:**

Both patients received levonorgestrel-releasing intrauterine system treatment and showed symptom improvement at 6-month follow-up. Pathology confirmed endometrial polyps and secretory phase endometrium, with no malignancy.

**Lessons:**

This study emphasizes the importance of NBI technology in identifying and diagnosing endometrial vascular dystrophy and its associated conditions, such as endometrial polyps. NBI provides clearer vascular imaging, aiding in precise targeted biopsies, thereby enhancing diagnostic accuracy and therapeutic efficacy. This research highlights the potential of NBI as a valuable tool in gynecological endoscopy for the diagnosis of rare endometrial conditions.

## 1. Introduction

Endometrial vascular dystrophy refers to abnormal vessels that are very tortuous and dilated and are rare under hysteroscopy. Endometrial vascular dystrophy was initially reported by Hamou in 1991.^[1,2]^ He classified endometrial vascular malnutrition into 2 types. The first was typified by uniformly distributed, miniature, dilated capillaries that appeared to be spiral vessels covering the whole endometrial surface. The second variation is characterized by meshed or branching capillaries, which are located directly beneath the basal endometrial layer. The phenomenon is still not well understood, with some scholars suggesting that the structures observed are actually glandular tissues filled with red blood cells,^[3,4]^ while others, including ourselves, believe these to be highly tortuous blood vessels. This manuscript presents a comparison of endometrial vascular dystrophy under white light and narrow-band imaging (NBI), further supporting the notion that the observed structures are indeed blood vessels.

NBI is an advanced optical technology that enhances the visualization of superficial mucosal vascular patterns, allowing for a more detailed observation of mucosal morphology and vascular architecture.^[5]^ Its application in gynecology, particularly in the diagnosis of endometrial pathologies, has been growing. We report 2 cases of endometrial vascular dystrophy observed under NBI (see Video, which shows the curved, spiral, and dilated small blood vessels observed under hysteroscopy with both white light and NBI), highlighting the potential of this technology in providing a clearer and more accurate diagnosis.

## 2. Case report

### 2.1. Case 1

**Figure 1.**
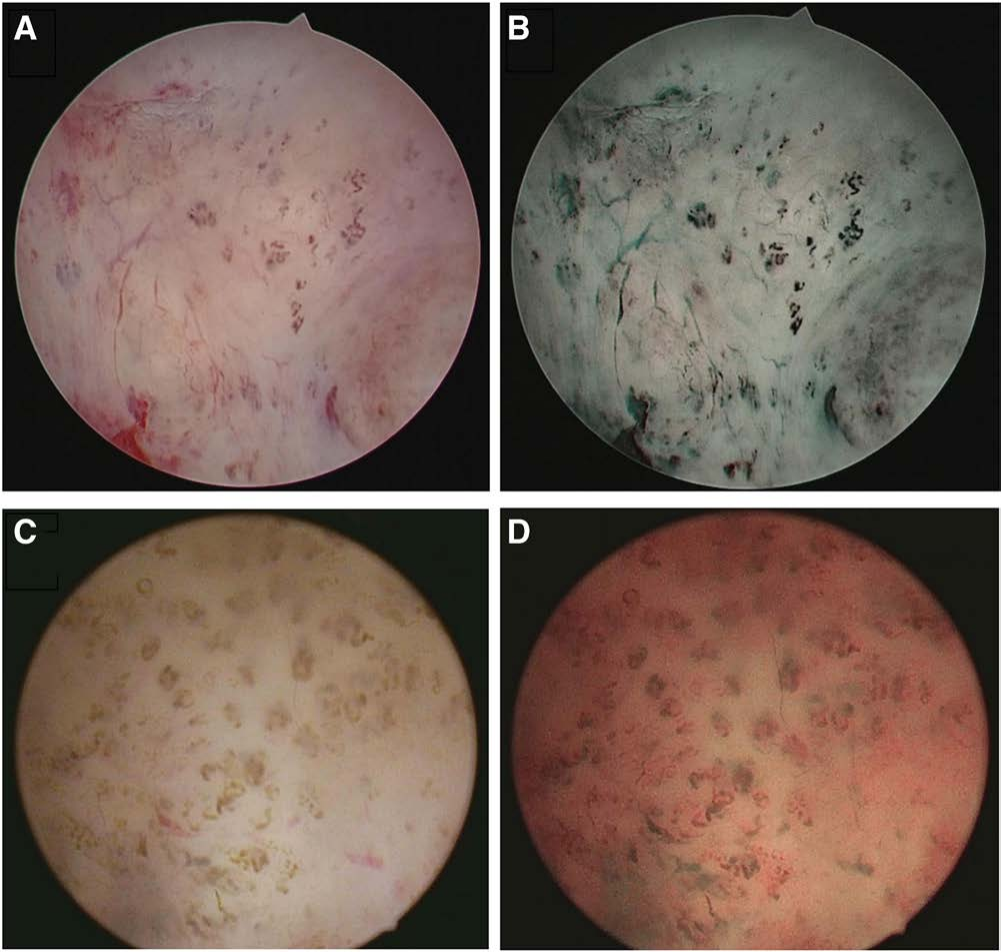
Endometrial vascular dystrophy manifests as a multitude of curved and dilated capillaries within the uterine cavity. Highlights the superior clarity of endometrial vascular structures achieved with NBI over standard white light, with vessels rendered distinctly visible under NBI. (A) and (C) were at white light. (B) and (D) were at NBI. NBI = narrow-band imaging.

A 32-year-old gravida 2 para 2 woman came to the hospital with vaginal bleeding for 8 days. She has experienced 2 months of intermittent bleeding. Her last menstrual period was 16 days ago, and before this, she had a regular 28-day menstrual cycle. She has no previous gynecological history of note, and her cervical screening test was 1 year ago and was negative. She has had 2 previous pregnancies with uncomplicated, normal vaginal deliveries. She has not been taking any contraceptives or special medication recently. In her medical history, there is no record of any specific illness, surgeries, or drug allergies. On speculum examination, a small amount of blood was found in the vagina, but the cervix appears normal. In the bimanual examination, the size of the uterus was normal without tenderness, and the ovaries and fallopian tubes could not be palpated. An ultrasound examination revealed an endometrial polyp. A hysteroscopy examination showed endometrial vascular malnutrition and endometrial polyps. A large number of curved, spiral, and dilated small blood vessels can be seen in the uterine cavity, and the blood vessels under NBI are clearly visible (Fig. 1A and B). The surgical process involves a fixed-point biopsy of the curved blood vessels and the removal of polyps. The pathological results showed endometrial polyps, with the endometrium in the secretory phase. No glands filled with red blood cells were found in the pathological section specimens. Clinical diagnosis: 1. Endometrial polyp 2. Endometrial vascular dystrophy. The patient was treated with levonorgestrel-releasing intrauterine system placement in the uterine cavity and was very satisfied with the follow-up 6 months later.

### 2.2. Case 2

A 47-year-old woman, with 2 pregnancies resulting in 2 live births (1 son and 1 daughter), presented for consultation regarding her menstrual concerns. She has a history of regular menstrual cycles lasting 4 to 5 days without any past episodes of dysmenorrhea, with her last menstrual period dated August 25, 2023. She has been experiencing increased menstrual flow and prolonged periods for the past 2 months. Specifically, her symptoms began 2 months ago without any identifiable cause, with her menstrual flow doubling from her usual amount and accompanied by blood clots. This heavy flow persisted for over 10 days before her period concluded. She did not experience any associated symptoms, such as dizziness or postexertional fatigue. Vital signs: temperature: 36.3°C, pulse: 66 beats per minute, respiration: 22 breaths per minute, blood pressure: 113/76 mm Hg. Gynecological examination revealed a small amount of blood in the vagina. Ultrasonography suggested the presence of an endometrial polyp. Outpatient hysteroscopy examination revealed several polyps within the uterine cavity. Tortuous blood vessels were visible on the fundus and both lateral walls of the uterus, which were more pronounced under NBI (Fig. 1C and D). The polyps were excised, and multiple biopsies of the tortuous endometrial vessels were taken. Pathology indicated endometrial polyps and secretory phase endometrium. After the surgery, the patient experienced drug-induced amenorrhea following the insertion of a levonorgestrel-releasing intrauterine system intrauterine device. A follow-up endometrial biopsy 6 months later showed drug-induced changes.

No approval of the research protocol is required, as our institutional review board does not mandate approval for case reports. We obtained written informed consent for the patient’s treatment, and the patient agreed that the relevant images and video materials could be used for publication in the article.

## 3. Discussion

Uterine endometrial vascular dystrophy remains extremely rare up to the present. Research on endometrial vascular dystrophy is still relatively limited, but several recent studies have provided us with some insights. Table 1 summarizes the recent studies on endometrial vascular dystrophy, including the number of cases, polyp cases, opinions on the nature of the structures, and histopathology results. From Table 1, it can be seen that there are different views among researchers on the nature of endometrial vascular dystrophy. Studies by Paoletti et al, Jiang et al, and Huang et al support the view that it is a vascular dystrophy, while studies by Bullón Sopelana et al,^[4]^ Lata et al,^[3]^ and Eiran^[6]^ believe that the structures observed are actually glands filled with blood, not abnormal blood vessels. As Sopelana et al^[4]^ observed convoluted secretory glands filled with blood in pathological sections. However, they were unable to explain how these red blood cells entered the glandular cavity. Jiang et al^[7]^ reported postoperative pathological sections did not reveal glands filled with blood. This difference may stem from a lack of understanding of the pathophysiological mechanisms as well as different interpretations of histopathological examination results. However, the exact mechanism is still unclear and requires further research for elucidation. This vascular distribution pattern is not only visible in the lining of the uterine cavity but also plays a crucial role in the early diagnosis of esophageal cancer, gastric cancer, and oral-pharyngeal lesions.^[8–10]^

**Table 1:** Summary of recent studies on endometrial vascular dystrophy.

Study	Year	Title	Case	Polyp cases	Nature of structures opinioned	Histopathology
Paoletti et al^[2]^	2012	Hysteroscopic Diagnosis of Endometrial Vascular Dystrophy	2	1	Considered vascular dystrophy	Secretory endometrium with stromal decidualization
Bullón Sopelana et al^[4]^	2019	Hysteroscopy: Endometrial Vascular Dystrophy	8	4	Not vascular dystrophy, but blood-filled glands	Secretory endometrium
Lata et al^[3]^	2021	Endometrial Vascular Dystrophy and Isthmocele: Hysteroscopic Visual Enigma	1	0	Not vascular dystrophy, but blood-filled glands	Secretory endometrium
Eiran^[6]^	2022	Hysteroscopy- Endometrial Vascular Dystrophy: Misnomer	1	1	Not vascular dystrophy, but blood-filled glands	Secretory endometrium with stromal decidualization
Jiang et al^[8]^	2023	Hysteroscopic Presentation of Endometrial Vascular Dystrophy: A Case Report	1	1	Considered vascular dystrophy	Secretory endometrium
Huang et al^[16]^	2023	Rare Case of Endometrial Vascular Dystrophy: Three Case Reports	3	1	Considered vascular dystrophy	Secretory endometrium

NBI is an optical image enhancement technique that filters the traditional red-green-blue broadband spectrum into a narrow band, increasing the contrast of superficial mucosal vascular structures and allowing for observation of mucosal morphology and vascular structure. NBI has been widely applied in the gastrointestinal, urological, and reproductive systems, improving the specificity and sensitivity of lesion diagnosis.^[11–14]^ In gynecology, NBI can better identify endometritis and endometriosis lesions, particularly by enhancing the accuracy, sensitivity, and specificity of detecting malignancies and precancerous lesions.^[5]^

Endometrial cancer often exhibits distinctive features under hysteroscopy, such as abnormal growths known as vegetations, significant disruptions in the normal mucosal architecture, and cerebroid-like appearances. The vessels may also display atypical characteristics, including irregularities in their size, distribution, and branching patterns. The use of NBI has demonstrated superior sensitivity in identifying both low-risk and high-risk hyperplasia. This enhanced detection capability is crucial for minimizing the likelihood of overlooking severe pathologies during hysteroscopic examinations and for enhancing the diagnostic accuracy of preneoplastic and neoplastic conditions.^[15]^

The 2 cases of uterine endometrial vascular dystrophy presented in this article highlight the distinct visualization of curved blood vessels within the endometrial layer when using NBI. A notable feature is the presence of a multitude of curved, spiral, and dilated small blood vessels within the uterine cavity, which provides a clear distinction from the vascular patterns observed in endometrial carcinoma. This distinction is pivotal for accurate diagnosis and differentiation from malignant conditions. A targeted biopsy at this site indicated secretory phase endometrium, with pathological sections not showing glands filled with red blood cells. Nearly all studies and pathological reports have identified secretory phase endometrium, suggesting a possible association with progesterone-related factors,^[2–4,7,16]^ although the exact mechanism remains unclear. Clinicians need to be aware of these abnormal blood vessels to determine the presence of cancer or precancerous lesions, as it influences subsequent treatment decisions and alleviates concerns for both physicians and patients. Fortunately, current reports indicate that uterine endometrial vascular malnutrition can regress spontaneously.^[2]^

Endometrial vascular dystrophy is a rare condition, and its diagnosis is often challenging. In this case report, the use of NBI technology allowed us to observe typical vascular structures that are difficult to discern under traditional white light. The application of this technology not only enhances diagnostic accuracy but also provides us with a deeper understanding of this rare condition. This study has several limitations that should be acknowledged. First, the rarity of endometrial vascular dystrophy means that our findings are based on a limited number of cases, which may not be representative of the broader population. This case report includes only 2 patients, and the conclusions drawn may be subject to bias due to the small sample size. Second, while NBI technology enhances vascular visualization, it is not without its limitations. The interpretation of NBI images still relies heavily on the experience and expertise of the clinician, which may introduce variability in diagnostic outcomes. Additionally, the pathophysiological mechanisms underlying endometrial vascular dystrophy remain unclear, and further research is needed to elucidate the exact nature of this condition.

## 4. Conclusion

This study confirms the value of NBI technology in diagnosing endometrial vascular dystrophy, providing a new tool for distinguishing this rare condition. Future research should further explore the application of NBI in gynecological endoscopic diagnosis to enhance diagnostic accuracy and efficiency.

## Acknowledgments

The authors would like to thank Ran Xiao’s help.

## Author contributions

**Conceptualization:** QiYin Zhou, WenDi Han, HaiHong Cui, JinCheng Huang.

**Writing—original draft:** QiYin Zhou, HaiHong Cui.

**Data curation:** WenDi Han.

**Writing—review & editing:** JinCheng Huang.
